# Rebuilding human resources for health: a case study from Liberia

**DOI:** 10.1186/1478-4491-9-11

**Published:** 2011-05-12

**Authors:** S Tornorlah Varpilah, Meredith Safer, Erica Frenkel, Duza Baba, Moses Massaquoi, Genevieve Barrow

**Affiliations:** 1 Ministry of Health and Social Welfare, Monrovia, Liberia; 2 Clinton Health Access Initiative, Monrovia, Liberia

## Abstract

**Introduction:**

Following twenty years of economic and social growth, Liberia's fourteen-year civil war destroyed its health system, with most of the health workforce leaving the country. Following the inauguration of the Sirleaf administration in 2006, the Ministry of Health & Social Welfare (MOHSW) has focused on rebuilding, with an emphasis on increasing the size and capacity of its human resources for health (HRH). Given resource constraints and the high maternal and neonatal mortality rates, MOHSW concentrated on its largest cadre of health workers: nurses.

**Case description:**

Based on results from a post-war rapid assessment of health workers, facilities and community access, MOHSW developed the Emergency Human Resources (HR) Plan for 2007-2011. MOHSW established a central HR Unit and county-level HR officers and prioritized nursing cadres in order to quickly increase workforce numbers, improve equitable distribution of workers and enhance performance. Strategies included increasing and standardizing salaries to attract workers and prevent outflow to the private sector; mobilizing donor funds to improve management capacity and fund incentive packages in order to retain staff in hard to reach areas; reopening training institutions and providing scholarships to increase the pool of available workers.

**Discussion and evaluation:**

MOHSW has increased the total number of clinical health workers from 1396 in 1998 to 4653 in 2010, 3394 of which are nurses and midwives. From 2006 to 2010, the number of nurses has more than doubled. Certified midwives and nurse aides also increased by 28% and 31% respectively. In 2010, the percentage of the clinical workforce made up by nurses and nurse aides increased to 73%. While the nursing cadre numbers are strong and demonstrate significant improvement since the creation of the Emergency HR Plan, equitable distribution, retention and performance management continue to be challenges.

**Conclusion:**

This paper illustrates the process, successes, ongoing challenges and current strategies Liberia has used to increase and improve HRH since 2006, particularly the nursing workforce. The methods used here and lessons learned might be applied in other similar settings.

## Introduction

Following fourteen years of civil war (1989-2003), Liberia's healthcare system was devastated. Most health professionals had fled or died during the fighting. In 1988, prior to the war, there were 3526 persons employed in the public health sector. By 1998, this number had reduced to 1396, with only 89 physicians and 329 nurses [1]. This paper introduces the historical and political context that led to the shortage of health workers in Liberia. It presents the important strides the health sector has made from emergency to development under the leadership of President Ellen Johnson Sirleaf (2005), focusing on the implementation of an emergency human resources (HR) plan to improve the numbers of qualified health workers. Using a recent census, a discrete choice experiment (DCE) and training institution studies, the paper evaluates the success in increasing the nursing workforce as well as the ongoing challenges around redistribution to hard-to-reach areas, training to improve skills, motivation and task-shifting to fill the gaps left by continuing physician and physician assistant shortages.

Health professionals began leaving Liberia to seek better opportunities when the country's economic growth began to slow during the late 1970s. In 1979, dissatisfaction over governmental plans to raise the price of rice led to protests in Monrovia. Seventy people were killed when military troops fired on protesters. Rioting ensued throughout Liberia and culminated with a coup by Samuel Doe in 1980. At this time and throughout the 1980s, as instability increased and the currency value decreased, high-level professionals continued to leave the country, creating large vacancies in the health system at all levels. This problem was only compounded when concessions (businesses operated under contract with business exclusivity within a defined geographical area) also pulled out of Liberia, taking with them their trained health workers.

In 1989 National Patriot Front forces, led by Charles Taylor, entered Liberia from Côte d'Ivoire and unseated the Doe government. By 1990 most medical specialists had left Liberia leaving only general practitioners. From 1989 to 2003, civil war resulted in a severely fragmented and incapacitated health system. As concessionaires and high-level workers left, non-governmental organization (NGO) emergency aid organizations began to arrive. The first to enter was Médecins Sans Frontières (MSF) in 1989. This began an NGO-centric health care system in which health facilities were dependent on external aid to function. By 2003, Liberia had 420 facilities (12 public hospitals, 32 public health centers, 189 public clinics, 10 private hospitals, 10 private health centers, 167 private clinics), 45% of which were being managed by NGOs and faith-based organizations (FBOs) [1]. Large numbers of displaced people moved into Monrovia, doubling the population and quickly outgrowing the city's capacity to provide health services with limited health workers and destroyed infrastructure. Community cohesiveness dissolved as members were displaced to Monrovia, neighbouring countries or new settlements along main roads.

Training institutions closed during fighting and re-opened during calm periods. By 2002, five of seven pre-war schools were operational: A.M. Dogliotti College of Medicine (physicians) was operational, but due to the collapse of the John F Kennedy teaching program it graduated only seventeen students between 1999 and 2002; Tubman National Institute of Medical Science (physician assistants, nurses, midwives, environmental health practitioners) graduated a total of 464 students between 1999 and 2002; from 2000 to 2002 Cuttington University College School of Nursing graduated 95 nurses and Mother Patern School of Health Science graduated 221 associate degree nurses. Phebe School of Nursing and Midwifery was operational but did not graduate students until 2003 [2]. The start and stop of education, limited educational resources and a lack of qualified professors in the country meant that few persons were able to go to school, fewer were able to complete it and none were able to match the quality of education received prior to the war. An Assessment of Health Training Institutions conducted by United States Agency for International Development (USAID) and the Ministry of Health and Social Welfare (MOHSW) in 2007 found that only Phebe School of Nursing & Midwifery and Mother Patern School of Health Sciences had the appropriate resources (textbooks, teaching laboratories, demonstration models, etc.) to provide a conducive learning experience [3].

For health workers that did remain in Liberia during the war, salary payments stopped and food became payment for work. In late 2003, Liberia signed the Comprehensive Peace Agreement in Ghana, ending the war and ushering in a transitional government supported by United Nations peacekeeping troops. In 2005, elections were held, and in 2006, Africa's first female president, Ellen Johnson Sirleaf, was inaugurated. By this time, there were less than 20 physicians, as compared to the 237 that had worked in the sector pre-war [4]. Nurses made up the majority of the remaining workforce. By 2006, there were 668 nurses (registered nurses, and licensed practical nurses) and 297 certified midwives. Together with an additional 1091 nurse aides, they provided the majority of primary care [2]. At the time that this paper is written, Liberia's health sector continues to face a severe shortage of qualified health workers across all cadres except nurses.

## Case description: rebuilding health human resources

### Establishing strong, coordinated leadership

By 2005, two years after the peace agreements were signed, the health sector was in disarray and dependent on more than $80 million of international humanitarian aid. Without oversight and coordination, this aid was distributed according to disparate donor priorities that did not necessarily match priority needs of the health sector [5]. As a result, the health system was barely functioning, with only an estimated 40% of Liberians able to access basic health services [6]. Following the inauguration of the Sirleaf administration in 2006, MOHSW initiated three reform actions in line with the national development priorities to strengthen healthcare delivery and outcomes in Liberia: (1) Build an experienced and visionary leadership team, divorced from political agendas; (2) Strengthen partnership and coordination to mobilize resources, align programs and harmonize all sector efforts; and (3) Develop and implement an evidence-based National Health Policy & Plan (NHP&P) to unify vision and direction for Liberia's post conflict health sector reform process.

The first reform priority was to build a strong leadership team with a shared vision for health reform. Ministry officials were appointed to their positions based on experience, academic qualifications, competence and good human rights records rather than political affiliations. The first action of the new MOHSW, in line with the second reform priority, was to coordinate and lead the many stakeholders in the sector. This resulted in the creation of two coordinating mechanisms: (1) the Health Sector Coordination Committee (HSCC), comprised of senior representatives of donors and partner NGOs who mobilize resources, advise the Minister and help guide the reform process and (2) the Health Coordination Committee (HCC), comprised of NGO/FBO service providers and MOHSW department officials to provide technical guidance on healthcare delivery.

With very limited information available, MOHSW developed the 2007-2011 NHP&P and focused on building management capacity at the central and county levels to enhance a coordinated approach. Donor funding was leveraged to support key management positions, including the establishment of the first MOHSW HR Unit. In December 2007, a HR Director was hired to coordinate all HR activities, including scholarships and incentives. Funded by the Civil Service Authority (CSA), the HR Unit is responsible for the development and oversight of HR policies and plans for the health and social welfare workforce, as well as to collect and disseminate HR data. Keeping with the NHP&P strategy of decentralization, funding was used to hire and train HR Officers to work as part of each County Health and Social Welfare Team (CHSWT) managing county worker recruitment, deployment and performance. Prior to the establishment of HR officers in each CHSWT, there wasn't anyone at the county level to feed data back to a central repository, enabling evidence-based HRH planning and management.

### Identifying Gaps

A critical next step to unify and drive health system reform was to understand the existing health needs and what gaps existed. To do this, MOHSW commissioned two integrated studies in 2006: (1) A rapid assessment, which sent enumerators to every county to identify the number, location and cadre of health workers; the number of functional health facilities; and the number of NGOs and FBOs; and (2) Community surveys to determine health priorities and recommendations for each region.

Findings highlighted the long-term adverse impacts of prolonged war on the health system. Curable diseases such as malaria, diarrhoea and acute respiratory infection emerged as the leading causes of morbidity and mortality. Maternal mortality, depending on the source, was estimated between 580 and 760 per 100 000 live births, while infant mortality was 157/1000 live births, and under-five mortality was slightly higher at 235/1000 live births [7]. Overall life expectancy at birth was 41 years [8]. Facility infrastructure was ruined due to looting or community displacement. Only 354 of the 550 pre-war facilities remained functional, of which 80% were operated by NGO or FBOs [9]. Without government oversight, NGOs and FBOs provided largely varying health services according to their own priorities. At the facility level, equipment had been destroyed or stolen; there was no electricity, little access to clean water and no communication network. Roads had been neglected, making many areas difficult to reach or, in some places, inaccessible during the rainy season. Without oversight, coordination and finances, most facilities were without needed drug and supply stocks.

Moreover, as most high-level professionals had left by the end of the war, a lack of management capacity at all levels and a shortage of qualified healthcare workers exacerbated each of these challenges. The rapid assessment determined the total clinical workforce (private, NGO and government) to be 3107 persons. Thirty-five percent of these were nurse aides and 30% were in the capital county of Montserrado due to accelerated urbanization. In 2006, with an estimated population of 3.2 million, Liberia had approximately 0.97 health workers per 1000 population, or 0.51 health workers per 1000 population if nurse aides were excluded [9]. There were a total of 965 nurses in Liberia: 402 Registered Nurses (RN), 297 Certified Midwives (CM), 214 Licensed Practical Nurses (LPN), 40 Nurse Anaesthetists, and 12 combined RN/CMs [9]. (An LPN received two rather than 3 years of formal training. The Zorzor LPN training program closed in 1991 due to the war and was not restarted in order to focus resources on training RNs. When referring to a nurse post-2006, it will be synonymous to RN.) Production of health workers was a complex challenge. Each of the remaining training institutions had significant operating challenges including ruined infrastructure, limited funding, lack of faculty and training capacity, overcrowded classes, outdated curricula, insufficient resources and no regulation [3].

Government salaries, set by the CSA, were low and did not regard grade, position or progression. Furthermore, government salary payment was consistently delayed and no incentives were paid to health workers deployed in hard to reach, underserved locations. These salary problems plus a lack of national benefits resulted in the migration of skilled staff to NGO facilities. Without HR information systems, one of the largest challenges became reconciling the payroll to identify and remove the high number of ghost workers (persons collecting pay but not working in the system or salaries paid to non-existent people).

### Moving forward: emergency human resources planning

Across Africa, countries that have experienced shortages of health workers like Liberia have adopted different strategies to address their health worker shortages. When Liberia's Emergency HR Plan was developed in 2007, several strategies from other African countries were considered. Similar to Ethiopia, Liberia considered creating a new cadre of health workers, called health care assistants, which would take a shorter time to train than nurses. This plan was modified to be a non-salaried program for community health volunteers, who currently provide education and treatment for diarrhoea-related illness in communities. This program will be scaled-up as more preventative and primary care training modules are developed.

Liberia borrowed a few principles from Kenyan and Malawian models, such as utilization of donor funds in Kenya to fill priority posts in the health sector, and the commitment of service required from beneficiaries of scholarships, stipends and housing in Malawi. Liberia's Emergency HR Plan 2007-2011 had four objectives: (1) Enhance a coordinated approach to HR planning; (2) Increase the number of trained health workers and their equitable distribution; (3) Enhance health worker performance, productivity and retention; and (4) Ensure gender equity in employment especially in management positions. Although targets were set for the recruitment and production of all cadres of health workers, nurses and midwives were prioritized as a means of addressing the high maternal and infant mortality rates in Liberia.

To increase the number of trained health workers, MOHSW took several measures to accelerate the development and recruitment of nurses and midwives. One measure was the standardization of salaries, which has been credited, by MOHSW Director of the Nursing and Midwifery Division, as the most important factor for the increase in the numbers of nurses hired by the government. This involved a review and standardization of salaries and allowances across the board in the health sector, in partnership with the CSA and Ministry of Finance, which effectively increased the pay of government health workers and ensured that health worker salaries were uniform within the Ministry as well as within NGOs. This helped stem the outflow of health workers from the public sector and also brought back health workers that might have left the health sector as a result of low salaries. Monthly salaries for nurses increased from 900 Liberian Dollars (US$ 13) to 7590 Liberian Dollars (US$ 108) in 2009 [Personal Communication: Baba, D. with MOHSW Director of Payroll, July 12, 2010].

Even with better salaries for health workers, MOHSW's ability to hire additional health workers was constrained by the dual challenge of limited resources and an employment ban in the public sector. The employment ban was one of the conditions Liberia agreed to in order to benefit from debt relief under the joint International Monetary Fund (IMF)- World Bank (WB) Bank Heavily Indebted Poor Country Initiative (HIPC). It was revised in 2007 to allow the government a moderate increase in minimum wage but continues to keep salaries low and impacts the ability of the government to hire new civil servants. The MOHSW HR Unit circumvented this employment ban by utilizing donor funds to boost its work force. This involved identifying priority positions together with donors and recruiting 'volunteer' health workers who were given an incentive payment in lieu of being placed on the government payroll. In 2009 the government of Liberia (GoL) allocated US$ 10,187,743 to the health sector. The personnel costs alone were US$ 6,962,709, amounting to 70% of MOHSW allocation from the government. With total MOHSW expenditures in the health sector amounting to US$ 23,524,554 in 2009, the MOHSW would have had a US$13.5 million gap were it not able to raise close to US$ 20 million from donors (Pool Fund, Global Fund, Earmarked Donor Funds, NGOs) [10]. As of June 2010, a total of 1748 nurses were receiving incentive payments from MOHSW and its partners. Additionally, all 11 senior ministry officials, 56 doctors and 23 pharmacists received incentives paid through donor funding [11]. These measures to increase the number of health workers working for the government without increasing its wage bill are considered to be stopgap measures. It is planned that these health workers will be absorbed on the government payroll as the economy continues to grow and allocations to the health sector increase.

Additional measures were taken by MOHSW to increase the pool of health workers that could be recruited in the future and improve distribution. Historically, medical education was free. However, during the war fees were introduced. In 2006, the government re-opened three rural training institutions and reinstituted free medical education to increase enrolment. Through the National In-Service Education Strategy, curricula for mid-level health workers were revised and standards of care introduced to improve pre-service training. From 2007 to 2011, GoL spent over US$ 335,000 to support student tuition at Liberia's government and private medical institutions. In-country scholarships have gone to students to become nurses, midwives, lab technicians, nurse anaesthetists and social workers. To date, 28 students have received international scholarships, funded by USAID, for program management or master degrees in public health. Sixteen of these students have completed their programs and returned to promoted health worker roles in Liberia. The remaining 12 are finishing their programs.

To improve distribution to hard to reach areas, the MOHSW HR Unit developed a regional incentive package to top up government salaries for persons working in hard-to-reach areas and re-introduced stipends with a bonding system for students (particularly student nurses). The bonding system requires health workers benefiting from stipends to serve the government in a hard to reach area for a period of time, usually corresponding to the length of their studies. Their certificate of graduation is given to them only after they complete the agreed upon service time in a hard to reach area. The first of these students should be graduating soon.

## Discussion and evaluation

### Increased numbers of health workers and their equitable distribution

In 2009, following the first full accreditation of facility provision of Liberia's Basic Package of Health Services (BPHS), staffing information was used by the HR Unit to identify facility gaps and deploy recent graduates from Phebe Nursing School and TNIMA. Twenty-three clinics without a required Officer In Charge (OIC) were prioritized to receive a PA or RN. Additional PAs and RNs, as well as CMs and Environmental Technicians were deployed to facilities with shortages. Table 1 shows the reduction in national staffing deficits based on the BPHS minimum staffing requirements from 2009 to 2010. Most notable is that the RN gap closed after these deployments, when all 46 identified positions were filled.

**Table 1:** Change in national health workforce 2009-2010

	2009 Deficit	2010 Deficit	Deficit reduction
Physician Assistant	46	31	33%
Registered Nurse	46	0	100%
Certified Midwife	263	207	21%
Laboratory Technician	32	34	-6%
Operating Theater Technician	90	80	11%
Anesthetist	77	21	73%

Sources: 2009 [16] and 2010 [17]

The Accreditation gave MOHSW its first look at national staffing since the development of the BPHS, however these numbers were subjectively reported by the facility OIC and not verified through employee records or visual confirmation. To improve information and begin strengthening HR strategies and planning, the MOHSW HR Unit completed the first national HR census in 2009. With support from the World Bank, the census confirmed the presence and qualifications of all accessible public and private facility staff, finding 8768 health workers, 4653 of which were clinicians. In 2010, with a population of 3.518,437, this equals 1.3 clinical health workers per 1000 population, far below the World Health Organization (WHO) recommendation of 2.2 health workers per 1000 persons in order to assure 80% of coverage of deliveries supervised by a skilled birth attendant.

While the overall ratio of clinicians to population remains low, w. Table 2 compares the number of workers per cadre in 2006 and 2009 against targets set in the Emergency HR Plan. In 2009, the percentage of the clinical workforce made up by nurses and nurse aides increased to 73%. During this time, the number of nurses more than doubled, the majority being RNs as the LPN program was discontinued. However, while the number of CMs increased by 28%, this fell far short of the Emergency Plan targets. Likewise, PAs, the interim strategy to offset the severe shortage of physicians, also fell dramatically short of the Emergency Plan targets. The overall sub-optimal production of CMs and PAs versus the significant increases in RNs suggests a lack of coordination with pre-service training institutions as well as inconsistencies in salaries and advancement opportunities. For example, an RN is paid more than a CM and is more likely to be placed as the OIC of a facility, thus receiving an increased monthly salary, US$ 75 greater than a CM.

**Table 2:** National stock of health workers by cadre as compared to Emergency Plan targets (2006 and 2009)

Cadre	2006 Rapid Assessment	2009 Emergency Plan Target	2009 Census	2009 Emergency Plan Shortfall	2010 Emergency Plan Target	2010 Emergency Plan Shortfall*
Physician	168	210	90	120	215	125
Physician Assistant	273	496	286	210	507	221
Nurse (RN/LPN)	668	567	1393	-826	595	-798
Nurse Aide	1091	n/a	1589	n/a	n/a	n/a
Certified Midwife	297	659	412	247	708	296
Dentist	13	n/a	23	n/a	n/a	n/a
Laboratory Technician	149	159	137	22	163	26
Laboratory Assistant	156	378	239	139	387	148
X-Ray Technician	25	60	22	38	62	40
Pharmacist	31	73	46	27	74	28

* Compared with the most recent data available: 2009 HR Census

Sources: 2006 [5] and 2009 [13]

**Figure 1:**
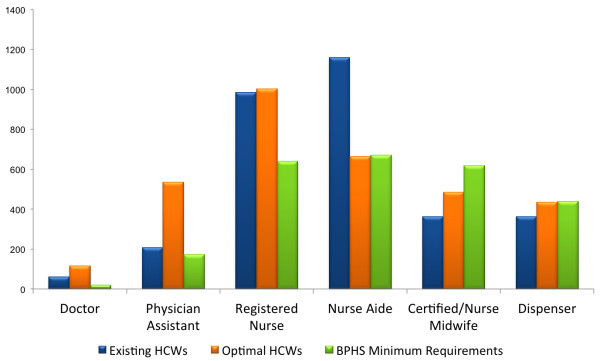
**National optimal workforce needs by cadre (2010)**. Source: [18].

As of 2009, the census showed that the numbers of physicians, RNs and nurse aides surpassed the BPHS minimum requirements. Recognizing that the requirements were four years old and set with limited sector information, the MOHSW HR Unit, with Clinton Health Access Initiative (CHAI) support, conducted a workforce optimization study to review minimum staffing requirements and calculate optimal workforce needs. The workforce optimization analysis utilized a demand-based model, which calculated the optimal number of health workers needed by cadre at health facilities based on service utilization rates and workload, obtained from the Health Management Information System (HMIS) database and worker interviews. Findings showed that while BPHS staffing requirements correctly identified the need for nurse aides and dispensers, the need for CMs was overestimated, and the need for physicians, PAs and RNs significantly underestimated. To inform priority setting, the study also identified the relative need for each of these cadres. Figure 1 shows the national optimal workforce relative needs by cadre.

While the nursing cadre numbers are strong and demonstrate significant improvement since the creation of the Emergency HR Plan, equitable distribution continues to be a challenge. The workforce optimization highlighted the concentration of nurses and health workers at hospitals and urban areas, to the disadvantage of health centers, clinics and rural areas. Table 3 shows the relative need of each health worker cadre by facility type. Nurse aides are the only cadre in which there is a surplus at each facility type. This surplus is minimal at the clinic level and increases significantly at the health center and hospital levels-most likely due to the informal task shifting-that happens when advanced clinical staff, such as PAs, are unavailable to do the tasks required at these facilities. Alternatively, CMs and RNs are concentrated at hospitals, leaving clinics and, in the case of RNs, health centers, severely understaffed.

**Table 3:** Relative need of cadres per facility type

	Physician	Physician Assistant	Nurse	Nurse Aide	Midwife	Dispenser
Clinic	n/a	500%	106%	-1%	151%	44%
Health Center	n/a	30%	56%	-36%	-15%	21%
Hospital	107%	29%	-52%	-82%	-58%	-53%

Source: [18]

Currently the MOHSW HR Unit, with WB and CHAI support, is conducting a training pipeline and costing analysis. These findings will identify costed intervention areas for Liberia's training institutions to meet the optimal workforce needs. As MOHSW works to increase the number of physicians and PAs, it is using findings from the workforce optimization study to formalize task-shifting opportunities. Once task-shifting plans are finalized to include appropriate remuneration, opportunities to redistribute nurses and midwives from areas with excess capacity to facilities/counties suffering severe shortages will be identified. This is particularly important considering that clinics are the primary point of care for the majority of Liberians, as most health centers and all hospitals are located in county capitals. If the number of surplus nurses found at hospitals were redistributed, it would meet the optimal need of all the clinics in Liberia and almost half of all health centers [12].

### Enhancing health worker performance, productivity and retention

#### Retention

In addition to being concentrated at hospitals, nurses are concentrated in urban areas, particularly the capital county of Montserrado. According to the 2008 National Census, approximately one-third of Liberia's population lives in Montserrado. Overall, 33% of health workers are in Montserrado, and of these, 6.8% were born in the county [13].

In order to comprehensively address retention in hard to reach areas, the MOHSW HR Unit and WB conducted a DCE for nurses in June 2010. The DCE quantitatively estimated how health workers value different aspects of their job in order to identify cost-effective policy options. Researchers spoke with a representative sample of nurses from a number of counties (notably three southern counties were excluded because they were too difficult to manage logistically) and questioned them on how they valued six aspects of their job: location, total pay, conditions of equipment, availability of transportation, availability of housing, and workload. The study recommended three policy interventions to increase retention of nurses in rural areas. The first is to recruit students from rural areas and expose all students to rural working conditions during their training. According to the DCE and corroborated by international evidence as described in the global policy recommendations "Increasing access to health workers in remote and rural areas through improved retention"[14], exposure to rural areas leads to a significantly higher willingness to work in those areas. Second, the most cost-effective option is to give US$50 bonuses to nurses working in rural areas. This would increase the percentage of nurses willing to work in the rural areas from 34% (baseline) to 49%. This is a similar increase that would occur if MOHSW improved equipment or provided housing, but at a much lower cost. Finally, the third intervention is to provide nurses in rural areas with transportation. Ideally, the DCE recommended combining this option with a US$50 bonus to substantially increase willingness to work in rural areas.

#### Productivity

Liberia has been using task shifting to increase service availability with limited HR since 1958 when the school for PAs was created to address the shortage of physicians in the country at the time. In recent years, however, the severe shortage of health workers at all levels has heightened the urgency of shifting tasks from highly trained providers to available staff with less training. As a result, throughout the war and in the years immediately following it, widespread, informal task shifting took place.

MOHSW has begun formalizing task shifting to ensure quality and safety. Focusing on the largest cadre of health workers, four areas are being task-shifted to nurses, midwives and nurse aides:

1. In addition to physicians and PAs, RNs and CMs will be trained to do emergency obstetric and neonatal care (EmONC) including caesarean sections at hospitals and health centers;

2. Nurse aides will be trained to be vaccinators across all facility types;

3. With only one psychiatrist in the country, nurses and nurse aides will be trained to provide mental health services.

MOHSW has created a new cadre of health worker, Nurse Anaesthetists, who will administer anaesthesia for minor operations at health centers and hospitals [Personal communication Frenkel, E. with Jessie Ebba-Duncan, MOHSW Assistant Minister for Preventative Services, July 11, 2010]. To do this, MOHSW is targeting both pre- and in-service training opportunities. Currently, MOHSW is working with training institutions to broaden the training of current students to include mental health and EmONC. For existing nurses, MOHSW offers training courses for nurses and nurse aides who are prepared to take on additional tasks. Finally, hospitals can apply for permission to train nurse aides in specific nursing services based on the needs of the facility. After receiving this training, the newly trained nurses will be permitted to perform those tasks only at the facility that trained them.

#### Performance

To improve performance, MOHSW has focused, to date, on in-service training and establishing strong leadership and oversight. With limited resources to invest in pre-service training and the need to improve the quality of services immediately, MOHSW created in-service training modules for the BPHS which every facility clinical worker is required to complete. To ensure dedicated HR leadership, the HR Unit was established and management performance improved through donor-funded technical assistance and international training opportunities. Two clinical supervision programs were implemented to ensure facility mentoring and monitoring. Each CHSWT is staffed with a Clinical Supervisor whose job it is to provide monthly supervision and assistance to each facility in the county. Additionally, central MOHSW teams are deployed to provide mentoring to the facilities once a year. Logistical challenges such as the constant disrepair of vehicles mean supervision does not currently happen as often as it should.

It has been increasingly recognized that implementing strong HR policy and management has to be at the core of any sustainable solution to health system performance [15]. Utilizing evidence from the studies described, the MOHSW HR Unit is currently finalizing the first HR Policy & Plan, which is expected to improve performance at all levels by clearly setting and communicating the standards. The BPHS Accreditation has helped to communicate service standards and measure progress against them. In doing so, it has ensured that each health worker has a clear understanding of what services should be provided at the facility. Setting clear expectations and evaluating performance at the individual worker level has been more difficult. Job descriptions are now standardized for each cadre, however they have not been broadly communicated to staff. For nurses, many of the tasks they are picking up through informal task shifting are not recognized in these descriptions. While a performance evaluation process was developed and is required, its practice is not widely implemented. Without increased compensation for additional tasks or years of service and no opportunities for advancement, motivation for nurses to improve performance is an ongoing challenge.

## Conclusion

Since the creation of the Emergency HR Plan in 2007, MOHSW has developed a strong management framework, improved HR coordination and significantly increased the number of nurses and midwives. Key interventions are responsible for these successes. First, strategically mobilizing donor funding and support to improve numbers and performance through training opportunities, salary incentives and technical assistance is credited as creating greater numbers of qualified nurses. Second, standardizing NGO salaries to match MOHSW pay amounts has stopped a large portion of outflow from the public to the private sector. Third, reopening training institutions and focusing on increasing skills through in-service training and mentoring has greatly reduced the number of nursing gaps at the facility level and increased nurses' ability to manage facility services that physicians and Pas would otherwise provide.

During this time, MOHSW has found that while strong leadership and uniform objectives are important, it is also necessary to admit weaknesses and ask for help when needed. Many of the standard international strategies to improve human resources such as continuing education, supervision and incentive payment do not consider Liberia's specific challenges. With the help of implementing partners and donors, MOHSW has found it useful to reject the international blueprint and develop strategies targeted to Liberia's unique challenges. Many of these challenges remain, particularly around regulation, payroll management, equitable distribution, retention of health workers in hard to reach areas and improving performance to impact the quality of services provided. In the last year, MOHSW has taken an evidence-based approach to understanding these challenges in order to define strategies for the first national HR policy and plan. Further work is needed to ensure population and utilization-based staffing norms, appropriate standardized salaries, improved training quality and production, opportunities for career advancement and a robust monitoring and evaluation system, critical to successful coordination. While the availability and reliability of MOHSW information systems has greatly improved, significant challenges remain for gathering and managing HR information. Following much work to develop CHT management capacity, MOHSW has recently begun installation of an HR software system that will enable continuous management of health worker employment, payroll and performance-based opportunities. In 2011 MOHSW plans to merge the HR Division and the Personnel Department, historically independent areas, to continue to streamline systems for improved coordination.

New initiatives to improve staff performance and motivation are underway, most notably the first county decentralization project and performance based financing. In 2010, MOHSW awarded the Bomi CHSWT US$ 2.2 million to fully manage and improve county health. A large part of this project is the work to determine the right package of financial and non-financial incentives in order to develop and maintain a qualified and motivated workforce. Health workers continue to be drawn to Monrovia for its housing, stronger school systems and easier work conditions. Currently, the CHSWT is exploring incentives such as weekend and overtime pay, staff housing and increased salaries to develop national strategies for retaining and improving staff in counties outside of Montserrado. Additionally, MOHSW has started using performance-based financing from its Pool Fund, and through partnership with the USAID-funded Rebuilding Basic Health Services (RBHS) project. Facilities meeting a defined set of indicators, including their BPHS Accreditation score, receive performance-based funding to use how they best see fit. This may be given out to staff or used to procure necessary items for the facility, etc. This process will be reviewed in 2011 to determine its impact. New available information, including the recently established catchment population database and community to facility distances will enable MOHSW to develop facility distribution and staffing norms based on population density and utilization. Finally, MOHSW is beginning to develop a quality management cycle. Rather than simply measuring the provision of BPHS services through the Accreditation, the quality of health workers' provision of services will be assessed.

## Abbreviations

BPHS: Basic Package of Health Services; CHAI: Clinton Health Access Initiative; CHAL: Christian Health Association of Liberia; CHO: County Health Officer; CHSWT: County Health and Social Welfare Team; CM: Certified Midwife; CSA: Civil Service Agency; DCE: Discrete Choice Experiment; EmONC: Emergency Obstetric and Neonatal Care; FBO: Faith-Based Organization; GDP: Gross Domestic Product; GOL: Government of Liberia; HCC: Health Coordination Committee; HEW: Health Extension Worker; HMIS: Health Management Information System; HR: Human Resources; HRH: Human Resources for Health; HSCC: Health Sector Coordination Committee; IMF: International Monetary Fund; LD: Liberian Dollar; LPN: Licensed Practical Nurse; MD: Medical Doctor; MOH: Ministry of Health; MOHSW: Ministry of Health & Social Welfare; MSF: Médecins Sans Frontières; NDS: National Drug Service; NGO: Non-Governmental Organization; NHP: National Health Plan; NHP&P: National Health Policy & Plan; OIC: Officer In Charge; PA: Physician Assistant; RBHS: Rebuilding Basic Health Services; RHP: Rapid Staffing Hire Plan; RN: Registered Nurse; TNIMA: Tubman National Institute of Medical Arts; USAID: United States Agency for International Development; WB: World Bank; WHO: World Health Organization.

## Competing interests

The authors declare that they have no competing interests.

## Authors' contributions

The work presented here was carried out in collaboration between all authors. All authors read and approved the final manuscript.
